# Modelling of Mouse Experimental Colitis by Global Property Screens: A Holistic Approach to Assess Drug Effects in Inflammatory Bowel Disease

**DOI:** 10.1371/journal.pone.0030005

**Published:** 2012-01-18

**Authors:** Johan Gottfries, Silvia Melgar, Erik Michaëlsson

**Affiliations:** 1 Department of Chemistry, University of Gothenburg, Gothenburg, Sweden; 2 Alimentary Pharmabiotic Centre, University College Cork, National University of Ireland, Cork, Ireland; 3 Department of Bioscience, Cardiovascular and Gastrointestinal IMED, AstraZeneca R&D Mölndal, Mölndal, Sweden; Charité, Campus Benjamin Franklin, Germany

## Abstract

Preclinical disease models play an important role in the establishment of new treatment paradigms, identification of biomarkers and assessment of drug efficacy and safety. However, the accuracy of these models in context of the human disease are sometimes questioned, e.g. due to trials failing to confirm efficacy in humans. We suggest that one reason behind this gap in predictability may relate to how the preclinical data is analyzed and interpreted. In the present paper, we introduce a holistic approach to analyze and illustrate data in context of one of the most commonly used colitis models, i.e. the mouse dextran sulphate sodium (DSS) colitis model. Diseased mice were followed over time along disease progression and by use of tool pharmacological compounds activating nuclear hormone receptors, respectively. A new multivariate statistics approach was applied including principal component analysis (PCA) with treatment prediction subsequent to establishing the principal component analysis model. Thus, several studies could be overlaid and compared to each other in a new, comprehensive and holistic way. This method, named mouse colitis global property screening, appears applicable not only to any animal modelling series of studies but also to human clinical studies. The prerequisites for the study set up and calculations are delineated and examples of new learnings from the global property screening will be presented.

## Introduction

Inflammatory Bowel Disease (IBD), Crohn's disease (CD) and ulcerative colitis (UC), are multifactorial, complex, lifelong diseases with a broad spectrum of manifestations. The pathophysiology of IBD is still unclear but it is well acknowledged that multiple factors, including genetic, environmental and immunological, contribute to the occurrence and perpetuation of the disease. Furthermore, the patients on occasion also present with extraintestinal manifestations, such as cholangitis, uveitis, peri-anal and oral lesions. The disease severity is quantified by grading symptoms, which are then compiled in a univariate fashion to generate disease activity scores, such as the Crohn's disease activity index (CDAI).

Evaluating the outcome of new drugs in a clinical study is traditionally performed by statistical univariate methods created to discover statistically significant improvements for the treated patient group, in an unbiased way. However, univariate methods need high object inclusion numbers to meet their significance dependency and, in addition, some correlations are of truly multivariate nature [1]. In terms of complex diseases such as IBD, there is also a risk of overseeing clinically meaningful treatment effects. Such treatment effects may be diluted and masked by unrelated phenomena that are involved in the score, but not in the targeted pathophysiology. On one hand, these approaches are obviously required for drug development, both from ethical and business aspects and the regulatory authorities delineate stringent guidelines (see FDA guidelines), which the industry follows for good reasons. The above perspective relates mainly to the later development phase, i.e. clinical phase III studies, Research and early development studies, on the other hand, could benefit from parallel approaches, frontloading biological or physiological relevance versus statistical significance. Several studies [2], [3], [4] have indicated that multivariate analyses provide more in depth knowledge than significance test statistics on the physiology and at the systems biology level, and could therefore be applied to the study designs. Such holistic methods can for instance describe how individual animals alter their physiological pattern when they develop disease or when they are subjected to pharmacological treatment.

In terms of preclinical models for IBD, more than fifty models have been described including genetically modified, chemically induced, adoptive transfer of T cells and also few spontaneous models (reviewed in [5], [6]). It should be noted that none of these models fully represent any form of human IBD. Rather, they can be viewed upon as mechanistic models illustrating different physiological and pathophysiological mechanisms occurring in the gastrointestinal tract. As such, the models have contributed greatly to our current understanding of the underlying mechanisms of gastrointestinal inflammation and disease pathogenesis. However, the complexity of gastrointestinal inflammation is seldom illustrated in these studies. Rather, there is a bias towards symptom scoring that are compiled into univariate composite indices. Similar to the human situation [7], these composite disease activity indices may not be informative for the severity of the local inflammation, and may therefore not be optimal endpoints for understanding the underlying mechanisms behind the disease.

For both ethical and scientific reasons it is always important to seek new ways to make the best use of any sacrificed animal, which will lead to higher accuracy in ensuing clinical studies [8]. In line with this thought we, by the current paper, want to present a new way towards a holistic and robust modelling of animal data, as exemplified on the murine dextran sodium sulphate (DSS) model of colitis. This has been achieved by a global property screening (GPS) approach, using a new way to set up data matrices for principal component analyses (PCA), applied to the characterization of the mouse model *per se*, but also to pharmacological treatments with small molecules.

## Results

The present study includes two independent series of experiments using DSS-induced colitis in mice and thus two ways of using multivariate modelling in experimental IBD. The first series represents a description of the disease process over time in two mouse strains and the second series how one of the mouse strains respond to treatments with new chemical entities. The PCA models were calculated by inclusion of healthy control mice in combination with DSS-treated and DSS-treated plus placebo-treated animals. The animals undertaking any intervention, e.g. drug administration, were not used for building the model, but subsequently predicted into the scores space using the established PCA model.

### Multivariate modelling of the disease dynamics in two genetic backgrounds

**Figure 1:**
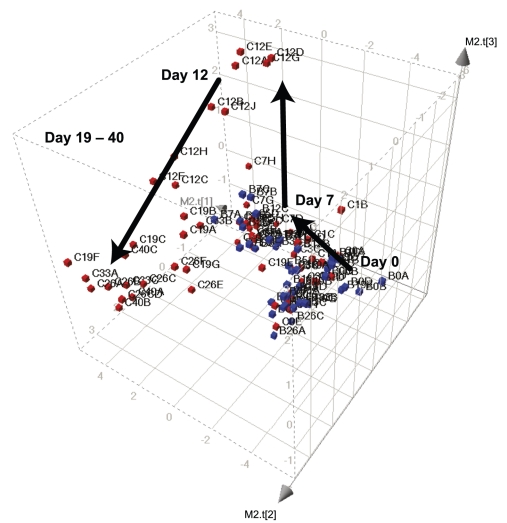
Multivariate projection of DSS-colitis development in BALB/c (Blue indicators, B) and C57BL/6 (Red indicators, C) mice. Each cube represents an individual animal. The number next to the cubes indicates the number of days since the initiation of DSS-treatment (from day 0 to day 40). Data were generated using a three-component principal component analysis (i.e. t1–t3 are plotted) based on the assessed variables according to Materials and Methods. Arrows indicate disease progression in C57BL/6 mice.

The first validation data set included DSS-treated C57BL/6 as well as BALB/c mice and the resulting PCA model provided models including 3 principal components. The mouse strain comparison between C57BL/6 versus BALB/c is depicted in Figure 1, previously shown to behave differently following exposure to DSS [9]. Indeed, the two strains behaved differently after the DSS challenge. This was observed by inspection of the individual animal positioning in the PCA scores plot. The PCA scores are generated by estimation of the object inter-related positioning in a scatter space comprising all collected biomarker data. The method implements each biomarker data as an individual and uniquely directed axis. In the example shown in Figure 1, 21 different biomarkers were assessed, thus generating 21 uniquely directed axes. Thereafter, the PCA least squares procedure was initiated, including the estimation of new vector dimensions adhering to minimized sum of squared distance from each data point to the generated vector, and thus the first PC is generated. In cases when the model needs more than a one-dimensional structure, a second and third and etc., component can be generated using the residual data (i.e. after removal of the data supporting the previously generated component). The validation for PCA model complexity was in the present study estimated by the cross-validation procedure as described in material and methods. Analysis of the present dataset yielded four significant PCs by the SIMCA P+ software. However, the fourth component explained less that 7% of the total data variance and comprised a negative contribution to Q^2^ (i.e. the predictivity as assessed by cross validation) and was therefore removed. The remaining three PCs explained 73% (i.e. R2 = 0.732 for the X matrix) of the data with Q^2^ = 0.47. The model scores, i.e. individual animal sample data scores, illustrating their time resolved reaction to the DSS treatment as assessed by biomarkers, are shown in Figure 1.

Evidently both strains were clustering together at day 0, i.e. all being healthy mice, after which the strain related property path diverted, which thus illustrated a tangible way towards mouse strain dependence to DSS response. Both strains revealed overlapping disease profiles throughout the initial week (d7). However, during the recovery phase the BALB/c mice moved, in the PCA projection scores system, in a close to inverse direction compared to the disease direction. After one week of recovery, these animals were apparently healthy and their individual position in the 3-dimensional score space were overlapping with that of healthy control BALB/c mice, illustrating complete recovery. C57BL/6 mice, on the other hand, did not recover towards a healthy phenotype, as seen in the PCA scores plot (Figure 1). Instead, they ended up in an orthogonal direction compared to the trajectory towards the healthy profile of the controls, but still different from the initial state induced by acute DSS-treatment, illustrating the chronic inflammation they suffered from.

In parallel, the influence from the primary biomarker variable correlation pattern on the property paths in the DSS experiment was inspected. This is achieved by use of the PCA inherent calculation of loadings, which are obtained by the assessment of the space angle between the direction of the original biomarker co-ordinate axis and the resulting PCs, respectively (PC1, PC2 and PC3 in the present example). A small space angle between a PC and a biomarker original axis indicates high correlation to the tested PC, and vice versa. Indeed the cosine of the obtained angle coincides with the correlation coefficient between the two axes. These estimates are called loadings (i.e. biomarker loadings in the present study), which are highly instrumental for entailing interpretation of observed individual animal trajectories and strain clustering seen in the PCA of data from the DSS induced colitis model (Figure 1).

Thus, we can conclude that the transition from health to acute disease (day 0 to 7) was characterised by the presence of blood in faeces and decreased biomarker loadings for colon levels of PGE_2_ in both BALB/c and C57BL/6 mice. In C57BL/6 mice, the transition from day 7 to 12 was characterised by high diarrhoea scores, increased weight loss, increased macroscopical signs of colitis and peak colon levels of IL-1β, CCL2, CXCL2/3 and CCL4. From day 19 and further on, the colon biomarker loadings for cytokine levels and macroscopical signs of colitis remained high. In addition, there were increased levels of CXL10 and IFNγ. Also, these time points were characterised by increased spleen weights and recovered body weights. Thus, for short term studies (up to 7 days) the two strains behave similarly and appeared equally relevant for drug intervention studies, whereas for chronicity studies the C57BL/6 mice should be used.

### Evaluation of new chemical compounds using PCA modeling

By pharmacological intervention we illustrate how the present approach can be used in drug discovery. In this example, a defined DSS-challenged mouse strain (BALB/c) was assessed for inflammatory biomarkers and other endpoints measured after treatment with three different nuclear hormone receptor agonists (i.e. LXR, PPARα and PPARγ). Nuclear hormone receptor agonists have been shown to possess anti-inflammatory action, possibly via mechanisms involving sequestration of and/or competition for NF-κB cofactors [10], [11].

**Figure 2:**
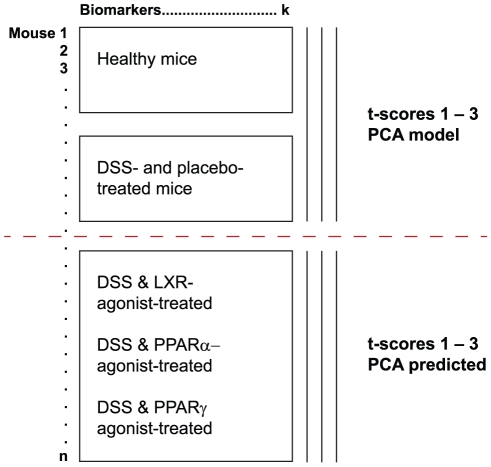
Schematic overview of matrix relation for the GPS calculations. The PCA model include healthy as well as placebo treated DSS-exposed mice. The distribution of these mice in the PCA model is shown in Figure 3A. The mice positioned into the PCA-model were treated with DSS- together with a LXR-agonist, PPARα-agonist or PPARγ-agonist. The positions of these mice are shown in Figure 3B.

**Figure 3:**
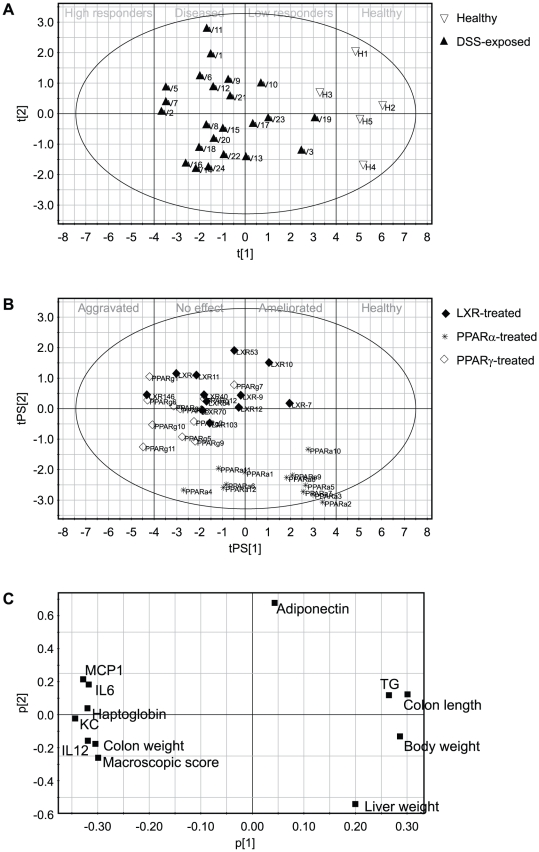
Evaluation of new chemical compounds using PCA modelling. A. Mouse colitis GPS model as generated from healthy control mice (open triangles) and placebo treated DSS-exposed mice (closed triangles) (see upper part of matrix in Figure 1). Based on the distance from the healthy control along the t(1) axis (i.e. the severity of the colitis) the mice are either classified as low responders, diseased or high responders. B. Mouse colitis GPS model including predictions for mice given nuclear hormone receptor agonists (dosing regimen as described in Materials and Methods), with receptor specificity as indicated (LXR (closed diamonds), PPARα (stars) and PPARγ (open diamonds)). Based on the position along the tPS(1) axis relative to the placebo-treated animals shown in Figure 3A, the effect of the treatment on the disease is either classified as ameliorated, no effect or aggravated. C. Individual variable correlation structure (i.e., loadings p1 versus p2 for PCA generated for objects in A) superimposable for interpretation of clustering in Figure 3A and B. The variables were plasma levels of haptoglobin, adiponectin and triglycerides (TG), colon levels of MCP1, KC, IL-6 and IL-12p40, colon weight, body weight, liver weight, colon length and macroscopic colon score. For example the PPARα-agonist treated animals in (B) are clustering in the lower end of principal component 2, due to high liver weight (and low adiponectin levels) and low principal component 1 due to reduced colitis.

First, a PCA-model was established using DSS- and placebo-treated and healthy control mice, respectively, with the aim to predict the data from animals receiving an additional compound treatment. This is possible for any PCA model given that the same variables, i.e. colitis biomarkers in the present study, are measured coherently. The data alignment aspect is depicted in Figure 2 and such prediction is straight-forward once a PCA model has been created. The expected advantages from this approach relates to standardization. Firstly, we provide a basic model that does not change in scale, biomarker loading correlation pattern towards the model PCs, or any other model property [12]. Secondly, when the PCA model is used for prediction of a pharmacological compound in treated animals (with or without DSS-pre-treatment) one can assess the cohort clustering and interpret their systems pharmacology positioning via the scores plot as compared to the healthy and DSS-treated cohort distributions (see Figure 3). However, in this step it is of outmost importance over time to include model animal objects to reveal any drift in the healthy and DSS treated animal clustering, be it for animal phenotype due to e.g. health status or response to DSS etc, or for drift in the analytical measures settings (data not shown). Thirdly, over time it is possible to combine any number of treatments and any comparison combination, including reference and tool compound treatments, for drug target and pharmacological mechanism investigation. By using the PCA prediction mode for new samples, i.e. predicting treated animal property positions into the initial model including the sample data from healthy and diseased animals, a robust model that facilitates interpretation between treatments over time is provided.

**Figure 4:**
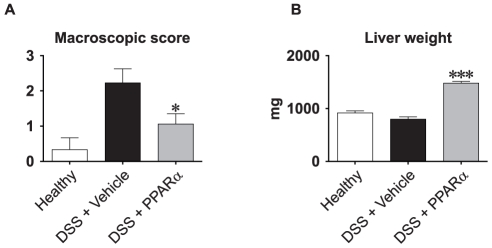
Univariate analysis of the effect of PPARα-agonist treatment on macroscopic colon scores (A) and liver weights (B). Macroscopic colon scores were assessed as described in material and methods. * p = 0.02; *** p<0.001.

The resulting systems pharmacology model on the present study is illustrated in Figure 3A, comprising 2 significant principal components. The reason for the lower model complexity compared to the previous example, where two mouse strains with DSS colitis were validated, was the restricted time used for the pharmacological intervention, i.e. 7 days, and thus the modelling of the “chronic” phase observed in C57BL/6 mice was omitted. Thereafter, the drug treated animals were positioned into the PCA model by prediction, illustrating the efficacies of three different nuclear hormone receptor agonists (see Figure 2 and Figure 3B). The biomarker and endpoint correlation pattern is depicted in Figure 3C. Figures 3A and B show the model based on healthy and vehicle-treated diseased mice. As shown in Figure 3A, mouse V3, V10, V17, V19 and V23 responded poorly to the DSS treatment (“low responders”), whereas the rest were classified as “diseased”, but not “high responders”. From the biomarker loadings (see Figure 3C), it was evident that all parameters except adiponectin and liver weight, were described by the first principal component, and this component was also what discriminated healthy from diseased mice. When the compound-treated animals were predicted into the model, it showed that treatment with the three NHR agonists generated three distinct patterns. Mice treated with the PPARγ agonist clustered towards the left-hand side along principal component 1 in the scores plot (i.e. negative scores, see Figure 3B), which represents higher plasma levels of haptoglobin (acute phase protein) and local markers of inflammation. Compared to PPARγ treated mice, LXR-treated mice comprised somewhat lower principal component 1 scores, demonstrating better anti-inflammatory efficacy. Finally, the PPARα-treated mice demonstrate the highest degree of disease amelioration of the three groups. However, the PPARα-treated mice also had low scores in the second component, due to high level biomarker loadings for liver weights in combination with low for plasma levels of adiponectin. From the loadings plot one can also conclude that the increased liver weight, which is a well-known effect by PPARα agonists [13], as well as the increased plasma levels of adiponectin, occurred independently from the anti-inflammatory action. Such interpretation can be drawn since adiponectin (and liver weight) loadings mapped orthogonally against the inflammatory biomarkers in the loadings plot (Figure 3C). Finding such uncoupled phenomena by orthogonal distributions of biomarker loadings illustrates an inherent property of multivariate analysis and unfolding the same pattern by univariate data analysis is less obvious. Rather, univariate analyses would, somewhat misleadingly, have suggested that liver weight increases occurs along with anti-inflammatory action (as illustrated in Figure 4).

## Discussion

In the present paper, we have exemplified how multivariate analysis can be used to illustrate complex disease processes in a holistic manner and also document effects of pharmacological interventions on experimental colitis.

One hurdle for using PCA-analysis in inter-experimental comparisons is the mathematical rotation along principal components (i.e. mirror images of t-scores) that can occur. Although a mirror image of the t score is mathematically as valid as the original t score, it precludes comparison with data based on the original t score However, by using a holistic global property screening (GPS) approach with fixed principal components as presented herein, such rotations along principal components are avoided. Thus, pharmacological models (Figure 1) and compound treatment (Figure 3) can be compared to each other without scores and loadings scale alterations or rotations. These procedures standardize the interpretations over time of sequential analyses and contribute to holistic knowledge gain, which is the core property of GPS modelling concept [12]. Thus, it provides a background template system, in which instilled perturbations, e.g. pharmacological compounds, strain shifts including mutations, and housekeeping studies for robustness testing, etc, can be monitored. In other words, the GPS will help any user to simplify any comparisons of phenotypic perturbation, be it from disease model aspects or compound distribution for disease amelioration purposes, as long as the data structure complies with each other's format regarding measurements of biomarkers and other characterizing variables (see Figure 2).

Extrapolating the surplus findings provided by the GPS approach in the present study makes it tempting to conjecture that similar modelling of human clinical phase II data [14] could provide new important and ethically relevant insights by global comparability. This would add to Chalmers' [15] well formulated arguments regarding the importance of transparency of clinical data for ensuing translational information exchange. Regarding the possible translation by GPS-modelling to clinical data one must keep in mind the importance of maintained ethical considerations and that study design must be strictly regulated. However, we envision that parallel application of multivariate methodology could lead to synergistic learnings in clinical data analysis, for instance by contextualisation of biomarker data. First, PCA is an excellent tool to find the unforeseen. Unlike traditional data univariate analysis, PCA is not hypothesis-driven, but a tool to discover correlations in an unbiased manner. Second, the holistic principles of PCA will identify synergistic processes that often occur in biological systems, which would not necessarily be unveiled using traditional null-hypothesis based, univariate data-analysis. Along the same line, the PCA will also readily pinpoint parallel, independent processes that may occur in biological systems, such as the effect of PPARα-agonism on liver weight vs. intestinal inflammation as shown in the present paper. This means that PCA could provide useful knowledge and be a means to deal with the publication bias in which inconclusive or negative data are not published [8], [15]. There are of course limitations with GPS. One considerable hurdle during implementation relates to the relatively abstract nature of the multivariate space. It can be challenging to understand why data, which at first glance appear to be un-structured, suddenly provide useful information and therefore should not be discarded. Secondly, the GPS-method is constrained to a pre-selected finite set of endpoints. As soon as one, or several variables are excluded or new variables are added into the data matrix (such as newly identified biomarkers), there is a risk of skewing and thereby limited predictive value of the model.

In conclusion, GPS modelling provides a good overview of data from complex measurements of biological systems, including holistic views on population distributions, definition of outlier individuals, and entailing model interpretation abilities to pinpoint serendipitous observanda, not the least by use of the additional GPS properties. This includes a potential for treatment stratification support [16], [17] due to the straight forward pattern recognition properties via scores and biomarker loadings interpretation with opportunities for generation of multivariate stratification criteria. Finally, given that both animals and clinical patients undergo intervention with the same compound, a data structure with inherent properties for concatenation between animal models and human disease might come closer in sight [4].

## Materials and Methods

### Ethics statement

The procedures described herein were approved by the local animal ethics committee in Gothenburg, Sweden (approvals 220/2002 and 62/2004). The mice were housed in an environment enriched with chewing sticks, nest material and egg cartons. During the experiment, the general condition of the mice was monitored at least twice daily and individuals that displayed a significantly impaired state of health, i.e. were inactive, had ruffled fur or were unwilling to seek chow and water, were euthanized.

### Mice, induction of colitis and tissue sampling

Specific pathogen-free female BALB/cOlaHsD or C57BL/6OlaHsD mice obtained from Harlan (Netherlands) were used. The mice were housed in groups of up to 8 mice per cage and acclimatised for two weeks before the start of the experiment. The mice were fed R3 pellet chow (Lactamin) and tap water *ad lib*. Addition of DSS to the drinking water was used to induce colitis as previously described [9], [18]. Briefly, a 3% (w/v) solution of 45 kD DSS (TdB consultancy, Uppsala, Sweden) was given for 5 days to C57BL/6 mice or 5% DSS for the indicated number of days to BALB/c mice. Fresh DSS was prepared daily to avoid bacterial growth in the solution. Healthy controls received normal tap water. Throughout the study, mice were monitored for symptoms (body weight, hunched posture, piloerection, and blood in the anus). During termination of the study, blood was taken by retroorbital puncture under isofluran anaesthesia and collected in EDTA-containing tubes, after which the mice were killed by cervical dislocation. The blood samples were centrifuged 3000 rpm for 10 minutes at 4°C, and plasma aliquoted and frozen at −80°C. The colon was dissected out, cut open, carefully rinsed and scored for macroscopical signs of inflammation (rigidity, thickness, ulcerations and oedema), yielding a maximal score of 10 [19], after which the distal 3 cm was divided and either frozen in liquid nitrogen (for cytokine analysis) or fixed in Zinc formaldehyde (for histology analyses) [9].

### Biomarker analyses

As a marker for systemic inflammation, the plasma levels of the acute phase protein haptoglobin were measured using a Cobas Bio centrifugal analyser with a haptoglobin reagent kit (TP801, Tridelta development). In the strain comparison shown in Figure 1, plasma was also analysed for the levels of IL-6, IL-12p40, TNF and CXCL1/KC using the xMAP technology developed by Luminex Corporation (Austin, Texas) as previously described [9].

To analyse local levels of inflammatory mediators, colon tissue was homogenised in PBS (Gibco, Invitrogen Corp UK) supplemented with 10% foetal calf serum and a protease inhibitor cocktail (Complete Mini, Roche Molecular Biochemicals), using a Homogenizer (Ultra Turrax T8, Tamro, Sweden). The homogenised material was centrifuged, 18000 rpm for 10 min, at +4°C, after which the supernatant was aliquoted and frozen at −80°C. To generate the data shown in Figure 1, the colonic levels of IL-1β, IL-12p40, IL-18, IFNγ, CXCL1/mouse KC, CXCL2/3-MIP-2, CXCL9/MIG, CXCL10/IP-10, CCL2/mouse JE/MCP-1, CCL4/MIP-1β, CCL5/RANTES and PGE_2_ were determined by ELISA according to the manufacturer's instructions (R&D Systems, UK and Cayman Chemical Company, MI (PGE_2_)). All cytokine data are expressed as pg/100 mg colon tissue.

### Treatment with nuclear hormone receptor agonists

BALB/c mice were exposed to 5% DSS in the drinking water for 6 days. The mice were orally treated by gavage with either 30 µmol/kg of the Liver X receptor (LXR)-agonist AZ-37328, 1 µmol/kg of the peroxisome proliferation activating receptor (PPAR)α-agonist AZ-63233 or 30 µmol/kg of the PPARγ-agonist AZ-15188, each group containing 12 mice. The compounds were suspended in hydroxypropylmethyl cellulose (Kleptose, Roquette) and administered at 10 ml/kg bodyweight. All treatments were performed once daily, starting one day before the addition of DSS and continued until the day before the termination of the experiment. Biomarkers in these experiments were colon levels of cytokines (IL-12p40, IL-6, MCP1 and KC determined by xMAP technology), and plasma levels of haptoglobin, triglycerides (Diagnostics GMBH, Mannheim Germany) and adiponectin (Linco Research Inc Missouri, USA). In addition, colon and liver weights were recorded and colons macroscopically scored for inflammation as described above.

### Data analysis and statistics

Data analyses were performed using PCA as implemented in the SIMCA-P software v. 11.0 and 12.0 (Umetrics, Umea, Sweden, see www.umetrics.com). PCA bilinearise a data matrix into scores and loading vectors, representing the objects and variables respectively [20]. Each endpoint (i.e. variable with its related loading) was, through-out all principal component analyses in the present study, given equal leverage to the ensuing model by the unit variance normalization procedure (according to the SIMCA P+ software default implementation) First the variance (i.e. the squared standard deviation SD) was calculated for each biomarker's data column and thereafter each individual datum value was divided by its biomarker variance value rendering a unit variance scaling. Thus the full data-set became re-scaled to comprise the standard deviation equal to 1, for each normalized biomarker data column, which in practise means an a priori equal chance of influence for each included biomarker measure independent on the initial raw data scale.

The relevant number of principal components (PC) was decided from the R^2^, Eigen value and loading structure, related to the respective component. Furthermore, cross-validation [21] was used as an additional measure for model judgement of proper model complexity.

Statistical significance shown in Figure 4 was calculated using the Mann-Whitney U-test in the software GraphPad Prism 4.0.
